# Joint care can outweigh costs of nonkin competition in communal breeders

**DOI:** 10.1093/beheco/arx137

**Published:** 2017-10-20

**Authors:** Kat Bebbington, Eleanor A Fairfield, Lewis G Spurgin, Sjouke A Kingma, Hannah Dugdale, Jan Komdeur, David S Richardson

**Affiliations:** 1 School of Biological Sciences, University of East Anglia, Norwich Research Park, Norwich, UK; 2 Behavioural Ecology and Physiological Group, Groningen Institute for Evolutionary Life Sciences, University of Groningen, Groningen, The Netherlands; 3 Faculty of Biological Sciences, University of Leeds, Leeds, UK; 4 Nature Seychelles, Mahé, Republic of Seychelles

**Keywords:** communal breeding, competition, cooperative breeding, offspring rivalry, relatedness, Seychelles warbler

## Abstract

Competition between offspring can greatly influence offspring fitness and parental investment decisions, especially in communal breeders where unrelated competitors have less incentive to concede resources. Given the potential for escalated conflict, it remains unclear what mechanisms facilitate the evolution of communal breeding among unrelated females. Resolving this question requires simultaneous consideration of offspring in noncommunal and communal nurseries, but such comparisons are missing. In the Seychelles warbler *Acrocephalus sechellensis,* we compare nestling pairs from communal nests (2 mothers) and noncommunal nests (1 mother) with singleton nestlings. Our results indicate that increased provisioning rate can act as a mechanism to mitigate the costs of offspring rivalry among nonkin. Increased provisioning in communal broods, as a consequence of having 2 female parents, mitigates any elevated costs of offspring rivalry among nonkin: per-capita provisioning and survival was equal in communal broods and singletons, but lower in noncommunal broods. Individual offspring costs were also more divergent in noncommunal broods, likely because resource limitation exacerbates differences in competitive ability between nestlings. It is typically assumed that offspring rivalry among nonkin will be more costly because offspring are not driven by kin selection to concede resources to their competitors. Our findings are correlational and require further corroboration, but may help explain the evolutionary maintenance of communal breeding by providing a mechanism by which communal breeders can avoid these costs.

## Teaser

Parents who raise their nestling in a communal nest alongside the nestling of other parents can reduce costly competition between offspring by providing more food. In the Seychelles warbler, we show that nestlings raised with a sibling have lower mass and survival than those raised alone, whereas nestlings raised with a nonsibling do not suffer these costs. Our results suggest that increased food provisioning can reduce competition among nonsiblings and facilitate the evolution of joint-nesting.

## INTRODUCTION

When parents provide simultaneous care to more than one offspring limitations on parental resources are expected to result in competition between offspring for resources (Mock and Parker 1997). Such offspring rivalry can greatly affect offspring fitness, either through direct disruption of resource acquisition or through investment in the development and maintenance of competitive traits (reviewed in Hudson and Trillmich 2008). As a consequence, offspring rivalry may influence parental decisions regarding the optimal level of investment for a given reproductive attempt (Trivers 1974; Parker et al. 2002).

In communally breeding species (also referred to as plural breeding in mammals [Jennions and MacDonald 1994] or joint-nesting in birds [Vehrencamp and Quinn 2004]), the offspring of multiple parents are reared in a joint nursery. While communal breeding may have thermoregulatory, safety, and energetic advantages in certain circumstances (reviewed in Vehrencamp and Quinn 2004), there are potential reproductive conflicts that must be overcome when offspring are reared in communal nurseries. As in noncommunally breeding species with multiple offspring, a communally-breeding parent can expect a reduction in the fitness of each of its offspring as a function of increasing brood/litter size but, unlike in noncommunal breeders, does not enjoy the reproductive benefit of having produced a greater number of its own offspring (Hodge *et al.* 2009). Additionally, the presence of additional, nondescendent offspring in the nursery may facilitate disease transmission (Saino *et al.* 1997) to the focal parent’s offspring, potentially further lowering the reproductive success of that parent. The extent to which offspring should compete with nursery-mates is partially determined by the benefit of acquiring resources and the cost of denying them to a related competitor (Parker 1989; Godfray 1995). Consequently, the lower within-brood relatedness inherent to communal nurseries (e.g., Williams 2004) provides a “battleground” for escalating offspring rivalry (Shen *et al.* 2010), potentially further increasing the cost of offspring competition for communally-breeding parents. However, explicit tests of the degree of offspring rivalry as a function of nest-mate relatedness, either in singular breeders or communal breeders, are largely missing.

There are 2 mediators of offspring rivalry that may play important roles in the evolutionary stability of mixed-relatedness nurseries in communally breeding species. Firstly, offspring rivalry arises as a result of limited parental resources (Mock and Parker 1997), but the increased number of caregivers in communal nurseries may increase per-capita resource availability to offspring so that costly competition is reduced (Shen *et al.* 2010); this may be particularly effective in systems where the ratio of carers to offspring is relatively high. Second, if parents have sufficient resources, they may attempt to mitigate the costs of competition for their own offspring by increasing prenatal investment to favor offspring growth and competitive ability, such as by producing heavier offspring (Hodge *et al.* 2009) or increasing prenatal provisioning of certain hormones (Schwabl 1996; Cariello *et al.* 2006). Thus, the extent of heightened offspring rivalry costs in communal nurseries depends on the balance between the negative effects of lower within-nursery relatedness and the positive effects of increased resource availability and prenatal provisioning.

In order to better understand the interplay between within-nursery relatedness, resource availability and offspring rivalry, we explored the costs of offspring rivalry in communal and noncommunal nurseries in a facultative communally-breeding passerine bird, the Seychelles warbler *Acrocephalus sechellensis*. In this species, most nests contain a single nestling (*singleton* broods) (87%, Komdeur 1994) but some nests contain 2 nestlings, which can either both be laid by the same female (*noncommunal* broods) or each be laid by a different (subordinate) female from the same social group (*communal* broods) (Richardson *et al.* 2001). Brood parasitism and egg-dumping are both entirely absent in this species (Richardson *et al.* 2001), and food is typically divided equally between nestlings in broods of two (Bebbington, Kingma, et al. 2016). By comparing nestlings raised with a competitor and singletons raised alone in the nest, we recently found that competition from a nest mate incurs body condition costs for all competitors and survival costs for the smaller of 2 nestlings (Bebbington, Kingma, et al. 2016). Given the inherent reproductive cost to raising 2 nestlings together, it is not clear how communal breeding remains stable in this system, nor indeed whether the costs of offspring rivalry vary between noncommunal and communal broods. Unlike many other communally breeding species, where infanticide is common (e.g., Trail *et al.* 1981; Macedo *et al.* 2001; Vehrencamp and Quinn 2004), communal Seychelles warbler nurseries are relatively peaceful; egg-rejection does not occur (Komdeur et al. 2005) and neither infanticide nor siblicide have ever been observed or suspected (Personal Observation). Previous work has shown that additional female parents in communal broods are, on average, not more related to the breeding pair than females who do not participate in the communal nest (Richardson *et al.* 2002). This result indicates that the parental costs of communal breeding are not mediated by preferentially sharing reproduction with a more related group member. Prior to the onset of breeding, females can interpret behavioral signals from other group members about their breeding intentions and hence predict whether their offspring will be competing with a less related nest mate (Cariello *et al.* 2006), which may allow them to adjust the competitive phenotype of their own offspring accordingly. However, females are likely to be restricted in their ability to preferentially invest in their own offspring after hatching (movement of chicks after hatching is likely to make imprinting difficult and selective feeding of nestlings has not been observed). Instead, females may be selected to produce a highly competitive offspring phenotype in order to mitigate the costs of offspring rivalry (Hodge *et al.* 2009). Importantly, unlike in many communally breeding animals, brood size is identical in communal and noncommunal Seychelles warbler broods, providing an ideal situation to test the absolute costs of offspring rivalry without the confounding effect of variation in the number of nestling competitors.

In this study, we use singleton nestling broods as a naturally-available comparison group to test for costs of offspring rivalry separately in noncommunal and communal Seychelles warbler broods. Specifically we test whether 1) noncommunal and communal broods differ from singleton nests in terms of per-capita resource availability to nestlings (including both spatial and temporal variation in territory-level food availability, as well as nest-level provisioning rates), 2) nestling pairs in noncommunal and communal broods differ in terms of relatedness, brood size asymmetry and total brood mass, and 3) nestlings in noncommunal and communal nests suffer different costs of offspring rivalry as measured through reduced body mass, telomere length (both these metrics are known to reflect condition and survival in this species: Richardson *et al.* 2004; Barrett *et al.* 2013; Bebbington, Spurgin, et al. 2016) and survival compared to singleton broods, and according to the relative competitive ability of each offspring. Our results indicate that the costs of offspring rivalry fall hardest on nestlings in noncommunal broods, who receive less per-capita food and have reduced body mass and survival to adulthood than those raised alone. This demonstrates that the potential costs of escalating competition between offspring of communal breeders can be mitigated by increased resource availability arising through communal care.

## MATERIALS AND METHODS

### Data collection

We sampled 247 nestlings from 203 nests, using long-term data from the Seychelles warbler database (Version 0.56.1) between 1995 and 2014 from the population of Seychelles warblers on Cousin Island, Seychelles (04°20′S, 55°40′E). During all major (June-September) and some minor (December-February) breeding seasons the entire population was censused and breeding adults were caught with mist nets. All birds were given a unique combination of color rings for visual identification and ca. Twenty-five microliters of blood were taken for sex determination, genotyping, and telomere analyses (see below). During each breeding season, all ca. 115 territories on the island were monitored for nesting activity. For all nests within reach, we sampled each nestling at between 10 and 14 days old, taking a small (15 µl) blood sample and measuring mass and tarsus length to the nearest 0.1 g and 0.1 mm, respectively. The time of day and month of catch were noted, since temporal variation in temperature and food provisioning may affect nestling mass. Where 2 nestlings were sampled in a nest (*n* = 42 nests), we assigned each as either the “A-offspring” (higher mass) or “B-offspring” (lower mass) as described in Bebbington, Kingma, et al. (2016). Each nest was then monitored until fledging or failure. Yearly censusing, combined with extremely low off-island dispersal (0.1%; Komdeur *et al.* 2004) and a high resighting probability (ca. 92%, Brouwer *et al.* 2006) means that individuals that were no longer seen across 2 consecutive seasons could safely be assumed to be dead, yielding highly accurate estimates of survival to adulthood (Brouwer *et al.* 2006; Barrett *et al.* 2013).

For 88 nests (43%) spanning all years of the study, we performed provisioning watches of at least one hour (mean duration ± SD = 64.3 ± 13.2 min) immediately before sampling the nestlings on ca. day 10 of the nestling period. From these data we determined the number of caregivers provisioning the nestlings, which can vary from 2 to 5, depending on the presence of provisioning subordinates (Komdeur 1994). Previous work has shown that provisioning rates observed at the same nest across the nestling period are moderately correlated (*r* = 0.45), suggesting that our observation regime is sufficient to produce a representative measure of provisioning rate at a given nest (Bebbington, Kingma, et al. 2016).

Communal broods are always provisioned by at least 3 caregivers (given that the extra female parent always provisions: Richardson *et al.* 2003), but the number of caregivers in singleton and noncommunal broods is variable. Using the provisioning watches, we also determined variation in resource availability in terms of per-capita provisioning rate (total provisioning rate per hour divided by brood size). There is also spatial and temporal variation in resource availability within the population, which we measured each year. During the period of fieldwork, we performed monthly counts of the number of insects found on the underside of leaves in 15 locations across the island. At the point of peak breeding (late July), we calculate foliage density in every territory on the island by recording leaf coverage at different height levels. Territory quality is then calculated as a function of insect abundance, foliage density and territory size, while island-wide food availability is calculated as the mean number of insects counted across all insect counts in a given season. Full details of these methods are described in Komdeur (1992) and Brouwer *et al.* (2006). Both territory quality and island-wide food availability were log transformed to provide a normal distribution.

### Molecular methods

DNA for sexing, telomere measurement and relatedness assignment was extracted using a DNeasy blood and tissue kit (Qiagen). Nestling sex was determined as described in Griffiths *et al.* (1998). We used quantitative PCR to obtain a relative measure of nestling telomere length (henceforth telomere length) as described in detail elsewhere (Barrett *et al.* 2013; Bebbington, Spurgin, et al. 2016).

Parent-offspring and nestmate–nestmate relatedness was calculated based on individual genotypes derived from a panel of 30 microsatellite loci previously developed for the Seychelles warbler (Richardson *et al.* 2001; Spurgin *et al.* 2014). To distinguish between communal and noncommunal broods, we first assigned all 2-nestling broods in territories with only one adult female present as noncommunal (egg-dumping does not occur in this species; Richardson *et al.* 2001; Hadfield *et al.* 2006). In territories with more than one resident female, we included all females as candidate mothers for each nestling and assigned maternity using maximum-likelihood estimation in MASTERBAYES 2.52 (Hadfield *et al.* 2006) with Wang’s (2004) genotyping error model, following the *MbG_Wang* method of Patrick *et al.* (2012). Genotyping errors were set to 0.0005—for full details see Bebbington, Spurgin, et al. (2016). Any nests where each nestling was assigned to a different female were considered “communal” (*n* = 8) and those where both nestlings had the same mother were “noncommunal” (*n* = 34). Relatedness (Queller and Goodnight’s *R*) between nestling dyads was calculated using Genalex 6 (Peakall and Smouse 2006).

### Statistical methods

**Figure 1:**
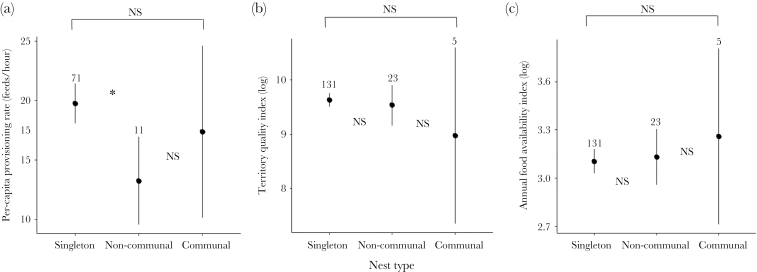
Differences in resource availability in terms of (a) per-capita provisioning rate, (b) territory quality, and (c) island-wide food availability between singleton and noncommunal, or singleton and communal broods in the Seychelles warbler. Dots and lines denote mean and standard error, respectively, numbers represent sample size per group. Significant (“*”) and nonsignificant (“NS”) differences between groups at *P* < 0.05 are displayed.

**Figure 2:**
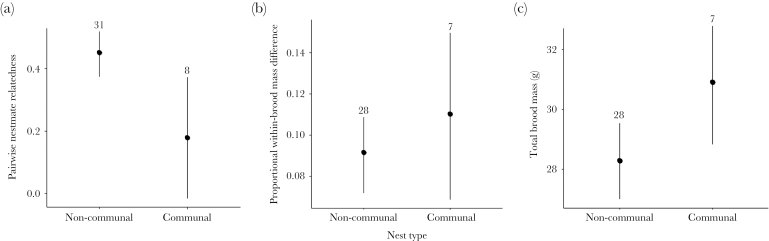
Brood-level differences in (a) relatedness, (b) nestling size asymmetry, and (c) total brood mass between noncommunal and communal nests (each with 2 offspring) in the Seychelles warbler. Dots and lines denote mean and standard error, respectively, numbers represent sample size per group.

**Figure 3:**
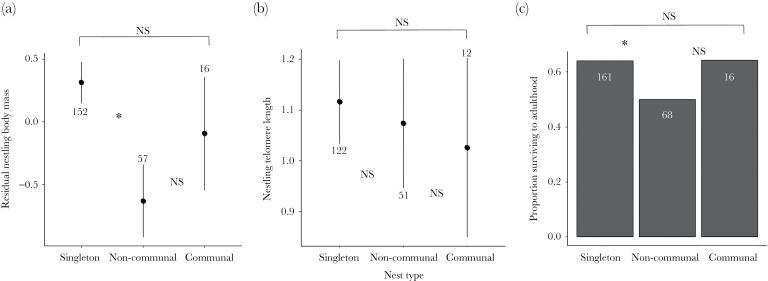
Differences in individual costs of offspring rivalry in terms of (a) residual body mass (controlling for tarsus length, sampling time and date), (b) telomere length, and (c) survival to adulthood, between singleton and either noncommunal or communal broods in the Seychelles warbler. In (a) and (b) dots and lines denote mean and standard error, respectively; in (b) bars represent mean values. In all panels, numbers represent sample size per group. Significant (“*”) and nonsignificant (“NS”) differences between groups at < 0.05 are displayed.

**Figure 4:**
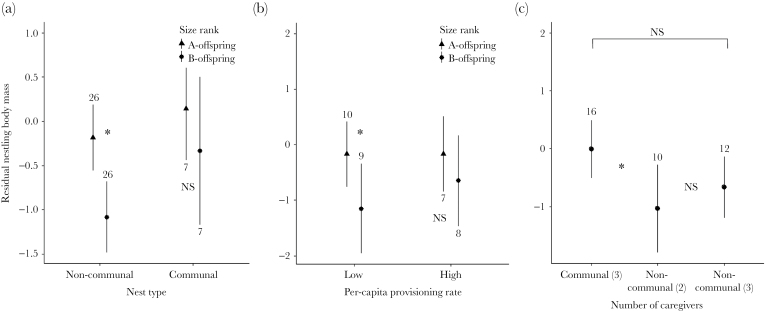
Interactions involving size rank and resource availability on residual nestling body mass (corrected for tarsus length, sampling time and date) in 2-nestling broods of the Seychelles warbler. (a) Influence of nest type on body mass according to size rank. (b) Influence of per-capita provisioning rate on body mass according to size rank. Note that per-capita provisioning rate was modeled as a continuous variable but grouped here or visual clarity. (c) Influence of additional caregivers in noncommunal nests. Noncommunal nests are split according to those that were provisioned by a helper-at-the-nest (caregivers = 3) and those that were provisioned only by the breeding pair (caregivers = 2) and both are compared to communal nests, which are always provisioned by 3 parents. Dots and lines denote mean and standard error respectively; numbers represent sample size per group. Significant (“*”) and nonsignificant (“NS”) interactions at *P* < 0.05 are displayed.

Unless otherwise stated, all analyses were conducted in R Studio (version 0.99.486, R Core Team 2015). We constructed generalized linear mixed models using the “lme4” package (Bates *et al.* 2015). Because we used multiple approaches and response variables to test our hypotheses, each of our analyses included different responses and predictor variables, not all of which were available for all individuals in the dataset. Sample sizes therefore vary between analyses; specific sample sizes for each analysis are therefore provided in Tables 1 and 2 and Figures 1–4. We checked for collinearity by calculating variance inflation factors for all our variables. *P* values were calculated using the Satterthwaite approximation in the R package lmerTest (Kuznetsova *et al.* 2015). In order to determine whether costs of offspring rivalry vary in noncommunal and communal nests when compared to nestlings raised alone, we report effects of nest type with reference to singleton broods. However, we also calculated differences between noncommunal and communal broods by changing the factor reference level; these contrasts are reported in the figures and in Supplementary Table S5. In order to maximize available degrees of freedom, we removed any predictors for which *P* > 0.1 to produce a minimal model. In Tables 1 and 2, we present the minimal model containing only significant predictors; the reported parameter estimates for these nonsignificant terms were obtained by reintroducing them individually into the minimal model and are displayed in Supplementary Tables S1–4.

**Table 1:** The effect of resource availability and brood-level differences between singleton broods and noncommunal or communal broods in the Seychelles warbler

Hypothesis	Response	Predictor	*F*	Estimate ± SE	*P*
Resource availability	Per-capita provisioning rate (*n* = 88)	**Nest type** ^**a**^	**5.28**		**0.02**
		** Noncommunal**		**−5.46 ± 1.96**	**<0.01**
		Communal		−1.08 ± 2.52	0.67
		**Observation time** ^**b**^	2.68		0.08
		Midday		0.50 ± 1.59	0.76
		** Late**		**3.49 ± 1.63**	**0.04**
		**Nest age**		**0.41 ± 0.18**	**0.02**
Brood-level differences	Relatedness (*n =* 39)	**Communal** ^**c**^		**−0.27 ± 0.09**	**<0.01**

*F* and *P* values for main effects of categorical variables are reported from an ANOVA. Significant predictors are highlighted in bold

Reference groups: ^a^“Singleton’. ^b^“Early”. ^c^“Noncommunal”.

**Table 2:** The effect of nest type (noncommunal or communal, compared to singletons) and additional predictors on 3 hypothesized costs of offspring rivalry in Seychelles warbler nestlings

Response	Predictor	*F*	Estimate ± SE	*P* value
Body mass (*n =* 225)	**Nest type** ^**a**^	**14.75**		**<0.01**
	**Noncommunal**		−**1.00 ± 0.19**	**<0.01**
	Communal		−0.53 ± 0.36	0.14
	**Tarsus length**		**0.74 ± 0.04**	**<0.01**
	**Catch time** ^**b**^	**3.68**		**0.03**
	**Midday**		**0.35 ± 0.17**	**0.05**
	**Late**		**0.52 ± 0.20**	**0.01**
	**Catch month**		**0.18 ± 0.06**	**<0.01**
Telomere length (*n* = 185)	**Tarsus length**		−**0.04 ± 0.02**	**0.03**
Survival to adulthood (*n* = 245)	**Tarsus length**		**0.27 ± 0.10**	**<0.01**
	Nest type^a^	2.41		0.09
	**Noncommunal**		−**0.78 ± 0.39**	**0.04**
	Communal		−0.47 ± 0.67	0.48

*F* and *P* values for main effects of categorical variables are reported from an ANOVA. Significant terms are highlighted in bold.

Reference groups: ^a^“Singleton”. ^b^“Early”.

### Resource availability

We first tested whether resource availability was different between singleton and noncommunal broods or between singleton and communal broods. We modeled per-capita provisioning rate as a Gaussian response and included nest type (singleton, noncommunal, or communal, where each nest constituted a single data point and singletons were the reference group), observation time (early: 0630–1100; midday: 1100–1500; late: 1500–1800 h) to account for variation in provisioning rates across the day and nest age (days since egg laying) as predictors. We included year of observation as a random effect to account for between-year differences. A second random effect of breeding pair identity nested in territory identity was included to account for repeat sampling of nests belonging to the same pair and territory across years.

To investigate differences in territory quality and island-wide food availability between nest types, we ran 2 separate logistic regressions: the first binary response was whether the nest was singleton or noncommunal, the second whether the nest was singleton or communal. We used log measures of territory quality and island-wide food availability as predictors in both regressions and included random effects of sampling year and breeding pair nested in territory identity to account for sampling of nests from the same year, parents or territory across the study period.

### Brood-level differences

Next we investigated brood-level differences between noncommunal and communal nests. Extra-pair paternity is high in the population (ca. 40%, Richardson *et al.* 2001), but it is unclear whether this varies between nest types and hence has the potential to affect the degree of offspring relatedness in noncommunal and communal broods. We therefore tested whether nestlings in noncommunal broods were indeed more related than those in communal broods using pairwise nestmate relatedness. We also tested whether brood size asymmetry (the proportion difference in mass between the A- and B-offspring) and total brood mass differed between noncommunal and communal broods. These latter 2 variables were calculated to test for differences in absolute brood mass, essentially reflecting parental productivity, between nest types (rather than mass controlled for structural size, which we investigated in a separate analysis). Nestling relatedness was modeled as a Gaussian response, with nest type (noncommunal or communal) as the single predictor. Brood size asymmetry (log-transformed) and total brood mass were modeled as Gaussian responses and we included nest age (days since egg-laying) alongside nest type as predictors. In this analysis, each breeding pair was only sampled once, but we included territory identity and year of sampling as random effects to account for repeat sampling of territories and years across the study period.

### Costs of offspring rivalry

We then tested whether offspring rivalry in noncommunal and communal broods confers costs in terms of reduced body mass, telomere length and survival to adulthood compared to singleton broods. We constructed mixed models that included nest identity (to account for common nest origin), year of sampling (to account for between-year environmental differences) and breeding pair nested in territory identity (to account for similarity in parental and rearing environments). In all models, we included nest type (singleton, noncommunal or communal, where singletons were the reference group) as a predictor. To investigate body mass (Gaussian response) we included tarsus length and its interaction with sex (to account for sex-specific mass-size scaling), along with time and month of sampling and nest age, as additional predictors. To investigate telomere length (Gaussian response), we included sex as an explanatory variable and also included nest age and tarsus length to account for potential differences in growth rate costs. These latter 2 variables are both related to the developmental stage of the nestlings but are not strongly correlated with each other (*R*^2^ = 0.07) and presumably describe different aspects of age-related variation in growth. To investigate survival to adulthood (binary response), we again included tarsus length and nest age. For all 3 response variables, we also included territory quality and island-wide food availability as additional predictors and tested for an interaction between these variables and nest type on offspring rivalry costs.

### Differential influences of competitive ability and resource availability

Lastly, we extended our analyses to investigate whether competitive ability and resource availability affected offspring rivalry costs differently for noncommunal and communal broods. To do this, we created separate models for body mass, telomere length and survival to adulthood, all of which included the random effects described above for the previous analyses (apart from breeding pair, which was unique for all nests in this analysis), along with any predictors that were significant in our initial analyses of offspring rivalry costs (see Table 2). Parameter estimates for these additional predictors were highly similar to those reported for the initial analyses and so are not reported here.

First, since the costs of offspring rivalry differ for the stronger and weaker of the 2 competitors (Bebbington, Kingma, et al. 2016), we tested for 2 interaction effects. To determine whether asymmetry in costs varies between nest types, we tested the interaction between nest type (noncommunal or communal) and size rank (A- or B-offspring), with the prediction that B-offspring may suffer more in communal nests due to lower nestmate relatedness. To test whether resource availability differentially influences the costs of rivalry for A- and B-offspring, we tested the interaction between size rank and per-capita provisioning rate across all 2-nestling broods, with the prediction that lower resource availability might more greatly affect B-offspring. Second, given that resource availability may differentially affect the costs of offspring rivalry in noncommunal and communal broods, we tested 2 further interactions across all 2-nestling (i.e., noncommunal and communal) broods. To test whether resource availability differentially affects offspring in different nest types, we tested the interaction between nest type and per-capita provisioning rate. To test whether variation in the number of caregivers influences offspring costs, we tested the relationship between offspring rivalry costs and the number of caregivers. Less than 5% of the broods in our dataset were provisioned by >1 helper so we considered helper presence or absence in binary terms. We modeled the number of caregivers as a 3-level factor: nonhelped noncommunal broods (2 caregivers), helped noncommunal broods (3 caregivers) and communal broods (always at least 3 caregivers), using communal broods as the reference group.

## RESULTS

### Resource availability

Per-capita provisioning rate varied over the day and increased with nest age (Table 1, Resource availability). Controlling for these factors, nest type had a significant effect on per-capita provisioning rate (Table 1, Resource availability). Per-capita provisioning rate was lower in noncommunal broods than in singleton broods, but per-capita rate to communal broods was not different to singletons—although the variance in the communal group was very high (Table 1, Resource availability; Figure 1a). Territory quality was not different between singleton and noncommunal broods, or between singleton and communal broods (Supplementary Table S1a, Figure 1b). The frequency of singleton, noncommunal and communal nests did not differ in relation to island-wide food availability (Supplementary Table S1a, Figure 1c).

### Brood-level differences

Nestlings were less related to each other in communal than in noncommunal nests (Table 1, Brood-level differences; Figure 2a). There was no difference in nestling size asymmetry between the 2 nest types (Supplementary Table S1b; Figure 2b) and size asymmetry did not vary with nest age (although there was a nonsignificant tendency for asymmetry to be lower in older nests, *P* = 0.06, Supplementary Table S1b). Total brood mass tended to be higher in communal broods, but this was nonsignificant (*P* = 0.08, Supplementary Table S1b; Figure 2c).

### Costs of offspring rivalry

Nest type had a significant effect on body mass (Table 2). Nestlings in noncommunal broods were of significantly lower body mass than those in singleton broods, whereas the mass of nestlings in communal broods were not significantly different to that of singletons (Table 2; Figure 3a). Neither territory quality nor food availability and neither showed an interaction with nest type (Supplementary Table S3). Nest age and sex adjusted for tarsus length (sex × tarsus length interaction) were also unrelated to nestling mass (Supplementary Table S2).

Telomere length decreased with tarsus length (Table 2) but did not vary with nest type: singletons did not have different telomere length to either noncommunal or communal nestlings (Supplementary Table S2; Figure 3b). Telomere length was not significantly related to nest age, offspring sex, island-wide food availability or territory quality (Supplementary Table S2) and neither food availability nor territory quality showed an interaction with nest type (Supplementary Table S3).

Nest type had a nonsignificant effect on survival to adulthood (*P* = 0.09, Table 2), suggesting that any differences between nest types are marginal. Nonetheless, nestlings in noncommunal broods were slightly less likely to survive to adulthood than those raised singly, but the survival of nestlings from communal broods did not differ from that of singleton broods (Table 2; Figure 3c). Nestling survival did not vary with nest age, island-wide food availability or territory quality (Supplementary Table S2), and neither food availability nor territory quality interacted with nest type (Supplementary Table S3). Survival increased with tarsus length (Table 2).

### Differential influences of competitive ability and resource availability

There was an interaction between nest type and size rank on nesting body mass: B-offspring were of lighter mass than A-offspring in noncommunal broods, but not in communal broods (β ± SE = −0.67 ± 0.28, *t*_30_ = −2.38, *P* ≤ 0.01, Figure 4a). No interacting effect of nest type and nestling size rank was observed for telomere length or survival to adulthood (Supplementary Table S4).

Across all noncommunal and communal broods, there was also an interaction between per-capita provisioning rate and nestling size rank on body mass: B-offspring were lighter than A-offspring when per-capita provisioning rate was low, but not when it was high (β ± SE = 0.05 ± 0.02, *t*_14_ = 2.29 *P* = 0.04, Figure 4b). This interaction was not significant for either telomere length or survival to adulthood (Supplementary Table S4).

No interaction was detected between per-capita provisioning rate and nest type: the influence of per-capita provisioning rate on body mass, telomere length and survival to adulthood did not differ between noncommunal and communal broods (Supplementary Table S4).

Compared to nestlings in communal broods (*n* = 16), nestlings in noncommunal broods with no helper (*n* = 10) were of lighter body mass (β ± SE = −0.81 ± 0.38, *t*_23_ = −2.21, *P* = 0.04, Figure 4c). Nestlings in noncommunal broods with a helper (*n* = 12) also tended to have lighter body mass than those in communal broods, but this relationship was marginally nonsignificant (β ± SE = −0.69 ± 0.34, *t*_26_ = −1.05, *P* = 0.06; Figure 4c). The number of caregivers had no effect on nestling telomere length or survival to adulthood (Supplementary Table S4).

## DISCUSSION

In this study, we determined whether nestlings in noncommunal and communal nests suffered costs of offspring rivalry and investigated the degree to which resource availability and competitive ability influenced those costs. We found that the 2 nestlings in noncommunal broods received less food per-capita than singleton broods and appeared to suffer body mass- and survival-based costs to offspring rivalry that appeared to be absent (or reduced) for the 2 nestlings in communal broods. Size rank played a more prominent role in determining the condition of individuals in noncommunal broods (vs. communal broods) and in all 2-nestling broods when per-capita provisioning rate was low versus high. Furthermore, the presence of a helper in noncommunal nests appeared to mitigate some offspring rivalry costs in terms of offspring body mass, which is known to predict offspring survival in this species. In combination, these findings suggest that resource availability, rather than within-nursery relatedness, is the principle driver of offspring rivalry costs in this species. However, it is important to note that while these different findings combine to form an apparently coherent pattern, they stem from a relatively small number of communal broods and thus should be interpreted carefully. In addition, the correlational nature of our findings cannot rule out potential confounds of parental quality, which would be better tested in other systems that can facilitate experimental work. Below we discuss the potential implications of these findings for our understanding of how offspring conflict can be resolved in communal-breeding systems.

Relatedness between nursery-mates has the potential to influence the degree to which parents disagree over the outcome of offspring rivalry (Parker 1989). Not surprisingly, nestlings in communal Seychelles warbler broods are significantly less-related to each other than those in noncommunal nests (Figure 2a), suggesting that there should be some degree of conflict between communally-breeding same-sex parents over the distribution of offspring rivalry costs within the brood. In noncommunally breeding species, parents often influence the distribution of rivalry costs, typically by increasing prenatal investment to, or initiating the earlier hatching of, preferred offspring (e.g., Mock and Plodger 1987). In a similar way, parents of communal broods should be selected to increase the competitive ability of their own offspring such that the majority of costs fall on other, unrelated offspring (Riehl 2010). The resulting conflict, where each parent would “prefer” for their coparents to bear the majority of offspring rivalry costs, has a clear parallel with sexual conflict over parental investment in species with biparental care. While the latter has received a great deal of both theoretical (Houston and Davies 1985; Lessells and McNamara 2012) and empirical (e.g., Schwagmeyer *et al.* 2002; Bebbington and Hatchwell 2016) attention, the resolution of parental conflict over offspring rivalry costs in communally breeding species remains a key point for future research.

Brood or litter size is assumed to be limited by, amongst other things, the availability of parental resources at the time of reproduction (Wilbur *et al.* 1974). Surprisingly, we found no evidence that the occurrence of either noncommunal or communal broods was related to increases in temporal food availability or greater territory quality (Figure 1). Resource availability is apparently also not more important for noncommunal than communal broods, which is surprising given that the reduced provisioning rate to noncommunal broods apparently reduces offspring fitness (see below); perhaps provisioning of noncommunal broods is limited not by absolute resource availability but by physiological constraints on the caregivers’ ability to supply that food. The prevalence of one-nestling broods and relatively long lifespan found in this species (Komdeur 1994) may mean that caregivers’ own future reproduction and survival prospects weigh heavier than resources in determining parental investment decisions (Trivers 1974).

Assuming that individual condition limits investment in individual offspring (e.g., Hodge *et al.* 2009), we envision 2 potential outcomes of conflict over the distribution of offspring rivalry costs in communal nurseries. Where extra, communally breeding parents are typically “subordinate” to a main breeding pair, such as in moorhens *Gallinula chloropus* (McRae 1995) and meerkats *Suricata suricatta* (Young *et al.* 2006), differences in social status and condition may lead to a natural competitive hierarchy in the nursery, similar to that found in many noncommunally breeding species (Mock and Parker 1997). Where extra parents are of the same social status with no clear dominance hierarchy, such as in the banded mongoose *Mungos mungo* (Gilchrist *et al.* 2004) and groove-billed anis *Crotophaga sulcirostris* (Vehrencamp 1978), the ability to invest in competitive offspring phenotypes should result in equal distribution of offspring rivalry costs within the nursery. We present 2 lines of evidence to support the latter outcome in Seychelles warblers. First, size asymmetry between nestlings in a brood was not significantly greater in communal than in noncommunal nests (Figure 2b), suggesting that nestlings of different mothers did not tend to be more divergent in terms of quality. Second, B-offspring appeared to pay a greater cost to offspring rivalry in noncommunal nests (at least in terms of body mass), while B-offspring in communal nests performed as well as A-offspring (Figure 4a). Therefore it seems likely that Seychelles warbler parents are unable to skew the costs of offspring rivalry away from their own offspring, but under what general circumstances this is the case is a highly interesting question that remains to be answered.

In noncommunal breeders, asymmetry within the brood probably evolves as a mechanism to ensure that at least some offspring are not exposed to the full costs of offspring rivalry (Mock and Parker 1997). However, noncommunal broods are also likely to exhibit a greater degree of hatching asynchrony than communal broods simply due to physiological constraints on egg-laying. In the Seychelles warbler, noncommunal broods are typically completed over 24 h (Komdeur *et al.* 2002) but communal broods can potentially be completed in one morning if both females lay on the same day (Komdeur 1994). Since hatching asynchrony would reduce the combined age of nestlings in noncommunal broods when compared to communal broods, an alternative explanation for our finding that noncommunal broods receive less per-capita food than communal broods is that the lower energetic requirement of younger noncommunal nestlings reduce the total amount of food parents need to provide. However, several lines of evidence lead us to reject this explanation. First, the nestling period is relatively long in the Seychelles warbler (17–19 days; Komdeur 1992) so 2 nestlings that differ in age by 1 day are unlikely to have fundamentally different total resource requirements than 2 of the same age. Second, we show that the proportion of size asymmetry between A- and B-offspring is not different between noncommunal and communal nests (Figure 2b), suggesting that any systematic differences in hatching asynchrony between noncommunal and communal broods do not have a detectable effect on offspring size differences. Finally, if hatching asynchrony is influencing size differences in noncommunal broods, we would expect a consistent difference in body mass between A- and B-offspring in these broods. The fact that B-offspring are only lighter than A-offspring when provisioning rate is low (Figure 4b) suggests that resource availability, rather than nestling age, drives the observed differences in body mass between A- and B-offspring in noncommunal broods.

The fact that B-offspring tend to suffer when provisioning rate is low suggests that when nursery-mates are forced to compete for more limited resources, they diverge in quality with respect to competitive ability. Similar patterns have recently been found with respect to milk transfer in spotted hyenas *Crocuta crocuta* (Hofer *et al.* 2016). It could be argued that the link between the high provisioning rate and apparent lack of offspring rivalry costs in communal nests is driven by some unknown factor that influences both of these variables. The fact that the number of caregivers seems to influence offspring body mass suggests that this is not the case: noncommunal nestlings who were provisioned by 2 parents were lighter than those in communal broods (3 parents), whereas the body mass of noncommunal nestlings with a helper was not significantly different from communal nestlings. This line of evidence adds support to the conclusion that offspring in communal nests do not suffer from sibling rivalry due to increased provisioning rate. However, it is worth noting that the addition of a third carer in noncommunal nests did not entirely mitigate the body mass cost for communal nestlings. This is likely due to nonbreeding helpers provisioning less than females who have produced offspring in the nest (see Richardson *et al.* 2002), but could also result from other, undetected differences between noncommunal and communal nests, such as egg quality (e.g., Cariello *et al.* 2006). By combining direct comparisons between noncommunally and communally reared nestlings and broader tests of variation in resource availability and competitive ability across all 2-nestling broods, we find evidence to support the hypothesis that any negative effects of reduced relatedness on offspring-level costs of rivalry are entirely mitigated by the additional food provisioning associated with communal breeding.

While we found evidence that body mass and survival differed with nest type, nestling telomere length did not differ between singleton, noncommunal and communal broods. It is worth noting that this may be due to our relatively low sample size in this analysis, but could also arise if the relationship between somatic costs and telomere length only manifests after some time. We generally sample nestlings on day 10 of the nestling period, which is just over half-way through the growth phase (when telomere loss tends to be greatest (Heidinger *et al.* 2012; Spurgin et al. 2017 ). It is possible that telomere length differences associated with varying costs of offspring rivalry would be more visible towards the end of the nestling period when, based on the patterns we find using body mass and survival, the most telomere shortening should have occurred in noncommunal nestlings. It is also possible that a measure of telomere loss, rather than relative length, would allow us to better detect costs of offspring rivalry. In the present study, we were unable to measure changes in telomere length during the nestling period due to issues with repeatedly disturbing nesting attempts in this rare species. However, aside from any inherited differences in telomere length (which appear to be relatively low in birds (Reichert *et al.* 2015), the measurement taken during sampling is likely to provide a reasonable approximation of telomere loss between hatching and sampling. In addition, nestling telomere length measured at a similar developmental stage has been shown elsewhere to vary according to brood size (Boonekamp *et al.* 2014) and also in relation to size rank (Nettle *et al.* 2015), suggesting that any differences in telomere loss between nest types should also be visible in this study. Perhaps the degree of differences between singleton, noncommunal and communal nests are not sufficient to cause differences in telomere length in the Seychelles warbler, but telomeres could potentially be used to measure differential costs of offspring rivalry in other facultatively communal breeders.

## CONCLUSIONS

Previous work has demonstrated that Seychelles warbler nestlings who are raised with a competitor have reduced body mass and suffer survival costs compared to those raised alone (Bebbington, Kingma, et al. 2016). Here, we show that both these costs are limited to nestlings reared in noncommunal broods and appear to be reduced or absent in communal broods. While relatedness between nestlings was considerably lower in communal than in noncommunal broods, the absence of within-brood competitive asymmetry or differential offspring rivalry costs in the former suggests that this competitive equality does not lead to escalated offspring rivalry costs. The patterns we report here rely on small sample sizes; validation of our findings in other facultative communal breeders is needed before any strong conclusions are drawn. However, the fact that resource availability appears to mitigate offspring rivalry costs more generally does support the hypothesis that escalated costs of competition among nonkin may be mitigated by the increased resource availability to communally-reared nestlings. We suggest that increased parental resources in communal broods, which likely arises as a consequence of a greater number of provisioning female parents, overrides any additional costs of increased competition between offspring of different parents. This finding could help explain how communal breeding can remain stable in the context of costly offspring rivalry and selfish genes.

## FUNDING

This work was supported by the Natural Environment Research Council (NE/H006818/1 and NE/F02083X/1 to D.S.R.). K.B. was supported by a Natural Environment Research Council PhD studentship.

## Supplementary Material

Supplementary Tables
