# Supplementary material for: Joint care can outweigh costs of nonkin competition in communal breeders
Source: Behav Ecol. 2017 Oct 20;29(1):169–78. doi: 10.1093/beheco/arx137 (PMC5873242; doi:10.1093/beheco/arx137)
Supplement: Supplementary Tables [file arx137_suppl_supplementary_tables.docx]

**Table S1** Parameter estimates for non-significant differences between singleton, non-communal and communal Seychelles warbler broods with respect to a) resource availability and b) brood-level differences.

| Hypothesis | Response | Predictor | Estimate ± SE | *P* |
| --- | --- | --- | --- | --- |
| a) Resource availability | Non-communal  vs singleton (*n* = 154) | Territory quality | -3.11 ± 2.30 | 0.18 |
|  |  | Food availability | 1.95 ± 2.19 | 0.37 |
|  | Communal vs singleton  (*n* = 136) | Territory quality | -4.56 ± 8.00 | 0.57 |
|  |  | Food availability | 0.66 ± 0.78 | 0.39 |
| b) Brood-level differences | Size asymmetry  (*n*  = 35) | Nest age | <-0.01 ± <0.01 | 0.06 |
|  |  | Communal^1^ | <0.01 ± 0.03 | 0.76 |
|  | Total brood mass  (*n* = 35) | Communal^1^ | 2.54 ± 1.39 | 0.08 |
|  |  | Nest age | 0.11 ± 0.20 | 0.60 |
| Reference group: | ^1^ ‘Non-communal” |  |  |  |

**Table S2** Parameter estimates for non-significant predictors of offspring rivalry costs in Seychelles warbler nestlings.

| Response | Predictor | *F* | Estimate ± SE | P - value |
| --- | --- | --- | --- | --- |
| Body mass  (*n* = 225) | Sex^1^ |  | 0.16 ± 0.14 | 0.25 |
|  | Nest age |  | -0.01 ± 0.02 | 0.46 |
|  | Territory quality |  | 0.07 ± 0.13 | 0.61 |
|  | Food availability |  | -0.19 ± 0.22 | 0.41 |
|  | Tarsus length * sex |  | 0.10 ± 0.07 | 0.18 |
| Telomere length  (*n* = 185) | Nest type^2^ | 0.21 |  | 0.81 |
|  | - Non-communal |  | -0.06 ± 0.08 | 0.49 |
|  | - Communal |  | -0.12 ± 0.15 | 0.43 |
|  | Sex^1^ |  | -0.05 ± 0.06 | 0.37 |
|  | Nest age |  | <0.01 ± <0.01 | 0.33 |
|  | Territory quality |  | <0.01 ± 0.06 | 0.99 |
|  | Food availability |  | -0.02 ± 0.12 | 0.88 |
| Survival to adulthood  (*n* = 245) | Nest age |  | < 0.01 ± 0.04 | 0.89 |
|  | Territory quality |  | 0.11 ± 0.30 | 0.70 |
|  | Food availability |  | 0.30 ± 0.53 | 0.57 |
| Reference groups: | ^1^ “Female”  ^2^ ‘Singleton’ |  |  |  |

**Table S3** Nonsignificant interactions between nest type and spatial and temporal resource availability with regard to the three measures of sibling rivalry cost in Seychelles warbler nestlings.

| Response | Predictor | Estimate ± SE | *P* |
| --- | --- | --- | --- |
| Body mass | Nest type * food availability | Non-communal 0.24 ± 0.42  Communal -0.10 ± 0.71 | 0.75  0.89 |
|  | Nest type * territory quality | Non-communal 0.02 ± 0.31  Communal 0.18 ± 0.46 | 0.95  0.69 |
| RTL | Nest type * food availability | Non-communal <0.01 ± 0.17  Communal -0.06 ± 0.32 | 0.98  0.84 |
|  | Nest type * territory quality | Non-communal -0.01 ± 0.13  Communal -0.16 ± 0.18 | 0.94  0.36 |
| Survival to adulthood | Nest type * food availability | Non-communal -0.32 ± 0.85  Communal -1.51± 1.43 | 0.71  0.29 |
|  | Nest type * territory quality | Non-communal -0.04 ± 0.68  Communal -0.72 ± 0.94 | 0.95  0.44 |

**Table S4** Nonsignificant interactions regarding the influence of competitive ability and resource availability on costs of sibling rivalry in non-communal and communal Seychelles warbler nests.

| Response | Predictor | Estimate ± SE | *P* |
| --- | --- | --- | --- |
| Body mass | Nest type * per-capita provisioning rate (versus non-communal) | 0.15 ± 0.09 | 0.11 |
| RTL | Nest type * size rank (versus non-communal)  Per-capita provisioning rate * size rank (versus A-offspring) | -0.06 ± 0.19  <-0.01 ± 0.02 | 0.75  0.66 |
|  | Nest type * per-capita provisioning rate (versus non-communal) | <0.01 ± 0.03 | 0.89 |
|  | Number of caregivers (versus communal) | Non-communal, no help: -0.08 ± 0.17 | 0.66 |
|  |  | Non-communal, help: 0.09 ± 0.17 | 0.62 |
| Survival to adulthood | Nest type * size rank (versus non-communal)  Per-capita provisioning rate * size rank (versus A-offspring) | 0.39 ± 1.42  0.09 ± 0.15 | 0.78  0.52 |
|  | Nest type * per-capita provisioning rate (versus non-communal) | 0.07 ± 0.18 | 0.71 |
|  | Number of caregivers (versus communal) | Non-communal, no help: 0.70 ± 1.45 | 0.63 |
|  |  | Non-communal, help: 0.07 ± 1.24 | 0.96 |

| Analysis | Response | Contrast | Estimate ± SE | *P* |
| --- | --- | --- | --- | --- |
| Resource availability | Per-capita provisioning rate | Communal | 4.58 ± 3.82 | 0.15 |
| Costs of offspring rivalry | Body mass | Communal | 0.47 ± 0.38 | 0.23 |
|  | Telomere length | Communal | 0.07 ± 0.17 | 0.68 |
|  | Survival to adulthood | Communal | 0.32 ± 0.70 | 0.65 |
| Differential costs of resource availability* | Body mass | Non-communal, 2 carers | 0.12 ± 0.47 | 0.79 |

**Table S5** Post-hoc model outputs showing the difference between non-communal and communal nests in each analysis (see main text for full details). Non-communal nests were the reference group in all cases, expect for the row marked * where non-communal nests with 3 carers was the reference group.
